# Rural–Urban Differences in Non-Local Primary Care Utilization among People with Osteoarthritis: The Role of Area-Level Factors

**DOI:** 10.3390/ijerph19116392

**Published:** 2022-05-24

**Authors:** Xiaoxiao Liu, Judy E. Seidel, Terrence McDonald, Nigel Waters, Alka B. Patel, Rizwan Shahid, Stefania Bertazzon, Deborah A. Marshall

**Affiliations:** 1 Department of Community Health Science, Cumming School of Medicine, University of Calgary, Calgary, AB T2N 1N4, Canada; xiaoxili@ucalgary.ca (X.L.); judy.seidel@albertahealthservices.ca (J.E.S.); alka.patel@ahs.ca (A.B.P.); 2 McCaig Bone and Joint Health Institute, University of Calgary, Calgary, AB T2N 1N4, Canada; 3 O’Brien Institute for Public Health, University of Calgary, Calgary, AB T2N 1N4, Canada; terrence.mcdonald@ucalgary.ca (T.M.); nwaters@ucalgary.ca (N.W.); rizwan.shahid@ahs.ca (R.S.); bertazzs@ucalgary.ca (S.B.); 4 Applied Research and Evaluation Services, Alberta Health Services, Edmonton, AB T5G 0B7, Canada; 5 Department of Family Medicine, Cumming School of Medicine, University of Calgary, Calgary, AB T2N 1N4, Canada; 6 Department of Geography, University of Calgary, Calgary, AB T2N 1N4, Canada; 7 Department of Civil Engineering, University of Calgary, Calgary, AB T2N 1N4, Canada; 8 Department of Environmental Science and Policy, College of Science, George Mason University, Fairfax, VA 22030, USA

**Keywords:** osteoarthritis, primary care utilization, rural–urban differences, geographically weighted regression

## Abstract

The utilization of non-local primary care physicians (PCP) is a key primary care indicator identified by Alberta Health to support evidence-based healthcare planning. This study aims to identify area-level factors that are significantly associated with non-local PCP utilization and to examine if these associations vary between rural and urban areas. We examined rural–urban differences in the associations between non-local PCP utilization and area-level factors using multivariate linear regression and geographically weighted regression (GWR) models. Global Moran’s I and Gi* hot spot analyses were applied to identify spatial autocorrelation and hot spots/cold spots of non-local PCP utilization. We observed significant rural–urban differences in the non-local PCP utilization. Both GWR and multivariate linear regression model identified two significant factors (median travel time and percentage of low-income families) with non-local PCP utilization in both rural and urban areas. Discontinuity of care was significantly associated with non-local PCP in the southwest, while the percentage of people having university degree was significant in the north of Alberta. This research will help identify gaps in the utilization of local primary care and provide evidence for health care planning by targeting policies at associated factors to reduce gaps in OA primary care provision.

## 1. Introduction

Rural–urban disparities in the prevalence of osteoarthritis (OA) have been reported at national and provincial levels in Canada, demonstrating significantly higher rates of OA in rural and remote areas compared to urban areas [1,2,3]. The population health burden of OA is expected to increase and reach one in four Canadians by 2040 [4,5] due to an aging population and rising rates of obesity [6,7]. Most importantly, patients suffering from OA—the leading cause of disability among adults—have a higher prevalence of co-morbidities, typically are older, and often have reduced mobility. As the first point of contact with the health care system, primary care physicians (PCP) play a central role in OA management through diagnosis testing, prescribing, coordinating specialty consultations, referrals to physiotherapy, and assigning home exercises. Early diagnosis, first-line treatments [8], and timely management of OA is necessary to slow the disease progression, reduce symptoms, and improve function [9,10].

The complex needs of patients with OA, especially in rural remote areas, necessitate the importance of enhancing care in the local community through the provision of access to local PCPs. Alberta Health (AH) identifies the utilization of non-local PCPs as one of the 13 primary health care indicators of community primary care need, which help identify the need for new or additional primary health services across all local areas throughout Alberta [11]. Rural and urban populations differ in their health status and health care utilization patterns due to the differences in community, spatial, and socio-economic characteristics between urban and rural areas [12,13]. The utilization of non-local PCPs is a complex outcome of many factors working together at the individual, family, and community levels. Factors affecting primary care utilization have been described in a theoretical model developed by Anderson [12,14], which includes predisposing factors, enabling factors and needs. Predisposing factors refers to population characteristics that cannot be altered to change utilization. Age and sex are two key disposing factors affecting OA health care utilization, as OA is more common in women and older populations [5]. In Alberta, the prevalence of OA among First Nations is twice that of non-First Nations [15]. Recent immigrants have a higher prevalence of arthritis than the non-immigrant population [16]. Need factors reflect disease characteristics and patients need of health services [14,17,18], for example, prevalence of OA and prevalence of comorbidities. Enabling factors refer to the resources and conditions that facilitate or inhibit the use of health care [14]. Spatial access to care, measured as travel time or straight-line distance between a patient’s home address and the location of health services they utilize [19,20], is a commonly reported enabling factor that may hinder or facilitate health care utilization. The literature has demonstrated the impact of spatial accessibility on health care utilization and health outcomes [21,22]; however, the results are not consistent among studies [23]—some reported negative association between longer travel time and worse health outcomes, some reported no relationship, and some reported positive association [23]. Socioeconomic status, another enabling factor to health care utilization, has been reported at both the individual and neighborhood level [24,25], suggesting that lower socioeconomic status is associated with a higher prevalence of OA; higher use of ambulatory care services, primary care physicians, and medications; and a longer period of hospitalization. However, studies suggested that the effect of spatial access to care on health outcomes may be modified after accounting for socioeconomic status [26,27]. Without controlling for socioeconomic status may overestimate/underestimate the true impact of spatial access to care on health care utilization and health outcomes [23].

Given the inherent spatial nature in the distribution of health care utilization as well as confounding factors [18], it is necessary to account for the importance of geographic confounding factors when health care utilization and associated factors vary by geographic location. Beyond individual-level factors, a spatial perspective has been increasingly adopted in health researches to explore potentially modifiable area-level factors that may impact health care utilization and health outcomes [28,29,30]. Geography was a significant barrier for indigenous peoples (First Nations, Metis, and Inuit [31]) in Canada due to difficulties in access to necessary health care services [32]. Our previous study examined the rural–urban disparities in the travel time to PCP for people with OA [33]. However, there is limited evidence on the extent to which patients with OA utilized non-local PCP care and if spatial access to PCP is associated with non-local PCP utilization while accounting for other confounding factors. Non-local PCP utilization is a key factor in identifying gaps in the provision of local care that is delivered to meet local needs and limit the use of non-local PCPs. Understanding factors that drive rural–urban differences in non-local PCP utilization will assist health care planning by targeting policies at associated factors and then reducing gaps in OA primary care provision.

In summary, non-local PCP utilization is an important primary care indicator identified by AH to support evidence-based health care planning decisions, which are especially important for people with OA who often experience reduced mobility. This study aims to identify spatial pattern of non-local PCP utilization, examine its association with spatial access to care and other area-level factors, and demonstrate if these associations vary between rural and urban areas. The results will help identify gaps in the provision of care and support the development and implementation of policies and health care planning to enhance care in local communities.

## 2. Materials and Methods

We conducted a cross-sectional observational study to examine the spatial pattern of non-local PCP utilization and associated confounding factors using administrative health data. Prevalent OA cohort in the fiscal year 2012/13 (1 April 2012–31 March 2013) were identified using a validated OA case definition [2,7,34]. Exploratory spatial analysis was applied to detect spatial pattern of non-local PCP utilization. Multivariate linear regression was applied to examine association of confounding factors with PCP utilization. Geographically weighted regression was applied further to understand how the association varies across rural and urban areas.

### 2.1. Prevalent OA Cohort in 2013

OA cases from 1 April 1994 to 31 March 2013 were identified using data from five AH administrative databases: Alberta Health Care Insurance Plan (AHCIP) population registry, the Physician Claims Database, the Discharge Abstract Database (DAD), and the Ambulatory Care Classification System (ACCS)/National Ambulatory Care Reporting System (NACRS) [7]. The criteria for our case definition included any patients who have at least one OA hospitalization or at least two OA physician claims within two years or at least two OA-related ambulatory care visits [2,7].

We identified 359,638 adult OA patients (≥18 years of age at diagnosis) from 1 April 2012 to 31 March 2013 who was identified as an OA case between 1994 and 2013 and was not dead or moved out of Alberta in 2013. A detailed description of OA case definition has been published elsewhere [2,7]. Patients were excluded if they did not seek PCP care in 2013 or if either patients’ postal codes or related providers’ postal codes were not recorded in the dataset.

### 2.2. Standard Geographic Areas

**Figure 1:**
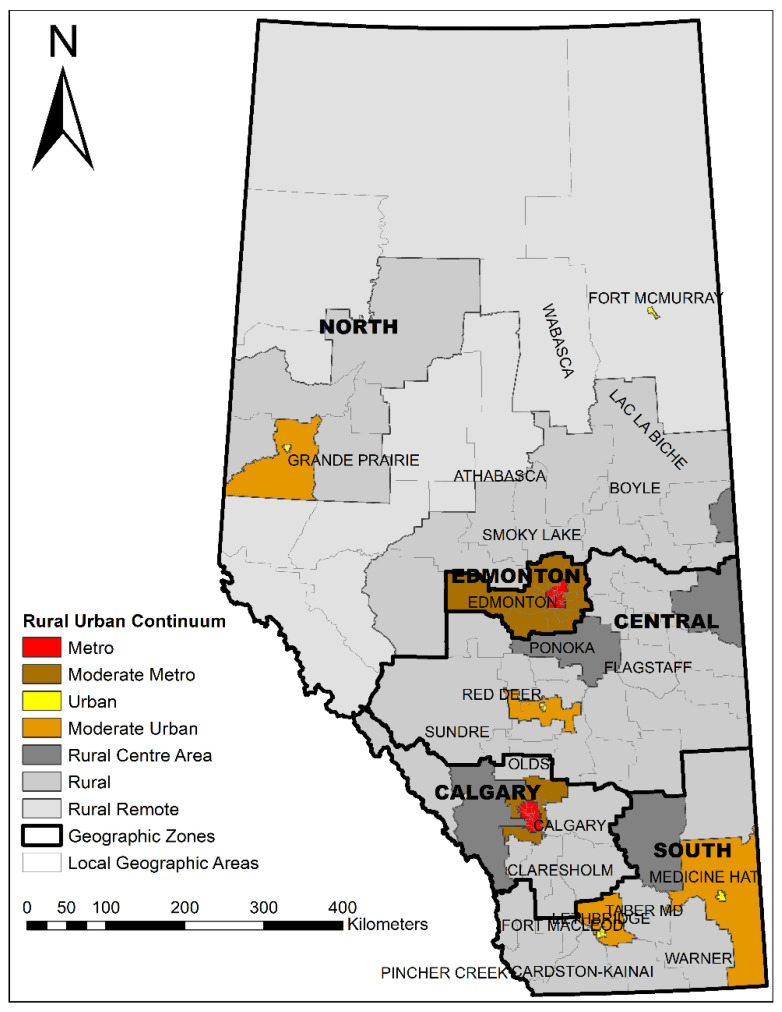
AHS standard geographic areas including a rural–urban continuum, 5 operational zones, and 132 LGAs.

Alberta Health Services (AHS) uses a set of standard geographic areas for planning, surveillance, monitoring, and reporting of population health, health outcomes, and health support services across Alberta [35] (Figure 1). The province is divided into five zones for operational purposes (North, Edmonton, Central, Calgary, and South Zones), which are further divided into 132 local geographic areas (LGA). AHS defines the rural–urban continuum using seven distinct categories (31 metro LGAs, 16 moderate metro, 9 urban, 5 moderate urban, 6 rural centers, 53 rural, and 12 rural remote LGAs) [35].

### 2.3. Non-Local PCP Utilization at the LGA Level

We extracted AH physician claims records that are associated with the identified OA prevalence cohort in 2012/13. The non-local PCP utilization was defined as the utilization of primary care outside of a patient’s home LGA. It was measured as the percentage of non-local PCP visits, that is, the number of non-local PCP visits divided by the total PCP visits (sum of local visits and non-local visits). Non-local PCP visits were identified by comparing the LGA of a patient’s residence to the LGA of the primary care clinic where care was provided. The visits were labeled as local visits if both patients and providers resided in the same LGA; otherwise, they were labeled as non-local visits. AH identifies the utilization of non-local PCPs as one of the 13 primary health care indicators of community primary care need, which help identify the need for new or additional primary health services across all local areas throughout Alberta [11].

Both patients and providers were geocoded using Alberta Health Postal Code Translator File (PCTF) [36]. Due to the confidentiality of providers’ IDs, we only obtained the postal code of a primary care clinic, that is, the location where the health services were provided. We obtained the residential 6-digit postal code of each patient from the AHCIP population registry dataset. We extracted the postal code of the provider’s practice location from the physician claims records. The PCTF file was used to locate each postal code within the 132 LGAs.

### 2.4. Independent Variables at LGA Level

We selected independent variables at the LGA level based on Anderson’s theoretical framework as well as data availability [14]. As defined by Anderson [12,14], factors affecting primary care utilization are grouped into three groups: predisposing factors, enabling factors, and needs. All the variables, their definitions, and data sources are listed in Table 1.

#### 2.4.1. Predisposing Factors

The predisposing factors used in this study were: age, sex, marital status, immigrant status, and First Nation ethnicity. We included two age variables: median age of OA patients visiting PCP in 2012/2013 and percentage of 65 years of age and older who live alone among the general population. Sex was represented as the percentage of female patients among total OA patients visiting PCP. Marital status was the percentage of female lone-parent families. Immigrant status was accounted for using the percentage of immigrants who arrived in the last five years. The First Nation ethnicity was calculated as percentage of First Nations with treaty status and Inuit among the general population.

#### 2.4.2. Enabling Factors

Enabling factors refer to the resources and conditions that facilitate or inhibit the use of health care, including median travel time to PCP visits within LGAs, ambulatory care sensitive conditions (ACSC), discontinuity of care, family income, and education attainment in this study.

Travel time in minutes was calculated as the drive time between a patient’s postal code and a provider’s postal code using an AHS-validated digital road network [37]. Travel time is considered as a proxy for access to a PCP. Given the skewed distribution of travel times, as discussed in pervious publications [33], we used the median travel time of patients with OA residing in the same LGA to represent local accessibility.

As an indicator for the robustness of primary care systems [38], ACSCs measure the acute care separation rate (per 100,000 population) over one year that may be avoided by providing appropriate primary care for the following seven conditions: angina, asthma, congestive heart failure, chronic obstructive pulmonary disease, diabetes, epileptic convulsion or seizure, and hypertension [11]. High ACSC indicates a problem in obtaining access to appropriate primary care. For example, the ACSC in rural Alberta in 2013 was 708.8 acute care separations per 100,000 population, compared to the rate of 388.2 in Alberta metro areas.

The discontinuity of care index describes the percentage of patients that are diagnosed as having any of the seven chronic conditions (hypertension, diabetes, asthma, chronic obstructive pulmonary disease, ischemic heart disease, congestive heart failure, and dementia) but have had no PCP visit within a three-year timeframe over the total general population. Lower values are preferable.

In this study, we included educational attainment—the percentage of general population with a university certificate, diploma, or degree at the LGA level. We also included income that was calculated as the average family income in each LGA and the percentage of family with after-tax low income, respectively.

The rural–urban status of patient residence was included in the analysis as a place indicator because place of residence is a determinant of health services use [39,40]. Based on the distribution of non-local PCP utilization by rural urban continuum (Appendix A) and considering the number of LGAs for subgroup analysis, we further grouped LGAs into two broad categories: broad rural (71 LGAs, including rural, rural center, and rural remote) and broad urban (61 LGAs, including metro, moderate metro, urban, and moderate urban). The two broad categories were used for the following multivariate linear regression analysis.

#### 2.4.3. Need Factors

Need factors reflect disease characteristics and patients need of health services, including age–sex standardized prevalence of OA and comorbidities (rate of people with three and more comorbidities per 100 population) in this analysis.

The variables of ACSC, discontinuity of care, and social economic indicators were obtained from the Primary Health Care Community Profile [11] from the Alberta Open government portal under the Open Government License—Alberta (https://open.alberta.ca/licence, accessed on 11 November 2020).

### 2.5. Statistical Analysis

#### 2.5.1. Exploratory Data Analysis

To explore the amount of clustering or dispersion of non-local PCP utilization, spatial autocorrelation was determined through global Moran’s I using a row-standardized spatial weights matrix based on six nearest neighbors [41,42]. Global Moran’s I index is an indicator for spatial autocorrelation and measures whether the spatial pattern of a variable is clustered, dispersed, or random. The Moran’s I value ranges from −1 to 1, where −1 indicates perfect dispersion, and 1 indicates perfect clustering. Statistical significance is checked using a Z-score and *p*-value with a 95% confidence level [41,42].

Gi* hot spot analysis [42,43,44] was applied to identify locations of statistically significant hot spot (high-value) and cold spot (low-value) of non-local PCP utilization. Detecting spatial pattern of non-local PCP utilization is important to understand the varying utilization pattern at local levels and provide evidence for health care planning and health resource allocation. LGA with a high percentage of non-local PCP utilization may be a statistically significant hot spot if it is also surrounded by other LGAs with high non-local PCP visits [42,43]. An estimated Z-score and *p*-value was calculated in each LGA, indicating areas with either high- or low-value clusters [42,43].

#### 2.5.2. Multivariate Linear Regression

A multivariate linear regression model [45] was applied at the provincial level (132 LGAs) and broad rural (71) and broad urban (61) LGAs, respectively, to explore the association of LGA-level factors to the non-local PCP utilization. Ordinary least squares (OLS) was applied to estimate the regression coefficients, aiming to minimize the sum of squared residuals. The presence of multicollinearity violates model assumption of no linear relationship between independent variables and leads to instability of model estimates [46]. Pearson correlation coefficients were calculated to measure multicollinearity between continuous-level independent variables. The Point-Biserial Correlation Coefficient was calculated to measure association between a continuous-level variable and a categorical variable. When two variables were highly correlated (>0.65 in absolute value) [47], only one was kept in the model. Full models were specified, each one containing alternative sets of uncorrelated variables. Using a backward, stepwise selection approach at each step, we gradually eliminated predictors that were not significantly associated with the response variable from the full models. Akaike information criterion (AIC) and adjusted R^2^ were used to compare and select alternative models. We applied the Shapiro–Wilk test for the assumption of normality of errors, the Breusch–Pagan test for the assumption of constant variance of residuals, the Moran’s I test for spatial autocorrelation in residuals, and the Lagrange multiplier test [48] for spatial dependence.

#### 2.5.3. Geographically Weighted Regression

Due to the presence of spatial dependence and spatial heterogeneity within spatial data, it is problematic to meet the assumption of independence and identical distribution of residuals [49]. Spatial regression techniques—geographically weighted regression (GWR) [50,51,52,53] and spatial autoregressive methods—yield more reliable models than standard regression techniques [54]. Spatial autoregressive models deal with spatial dependence, resulting in constant association between independent and response variable across the study area. GWR deals with spatial heterogeneity, accounting for spatially varying associations between independent and response variables. GWR is of interest in this study given the distinct health care pattern between rural and urban LGAs and the varying contributing factors. GWR captures the spatially varying structure and minimizes the variance caused by spatial heterogeneity. GWR computes as many local regressions as the number of LGAs in the province. Each local regression is performed on a subset of neighbors around the LGA of interest. We used a Gaussian kernel with adaptive distance to select the optimum neighbor size, which is determined by minimizing the cross-validation scores or the corrected Akaike information criterion (AIC) values [55]. Global Moran’s I test was applied to assess the spatial autocorrelation in the residuals. Variance inflation factors (VIF) were calculated to examine local multicollinearity in GWR models [56].

All the analyses were conducted using ArcMap 10.8 and R 4.0.2. Ethics approval for this project was provided by the Conjoint Health Research Ethics Board at the University of Calgary (REB13-0100).

## 3. Results

### 3.1. Exploratory Spatial Data Analysis

Among our cohort of 359,638 patients with OA in 2012/13, we identified 170,342 patients who accounted for 577,899 PCP visits (3.4 visits/patient). Out of the 170,342 patients, 28% resided in broad urban areas accounting for 30% of total visits compared to 72% of rural patients accounting for 70% PCP visits. The overall PCP utilization rate was 11% higher in broad rural areas than the corresponding rate in broad urban areas (3.64 vs. 3.29 visits/patient). The utilization of non-local PCP in broad rural areas was 26% compared to 61% in the broad urban areas.

**Figure 2:**
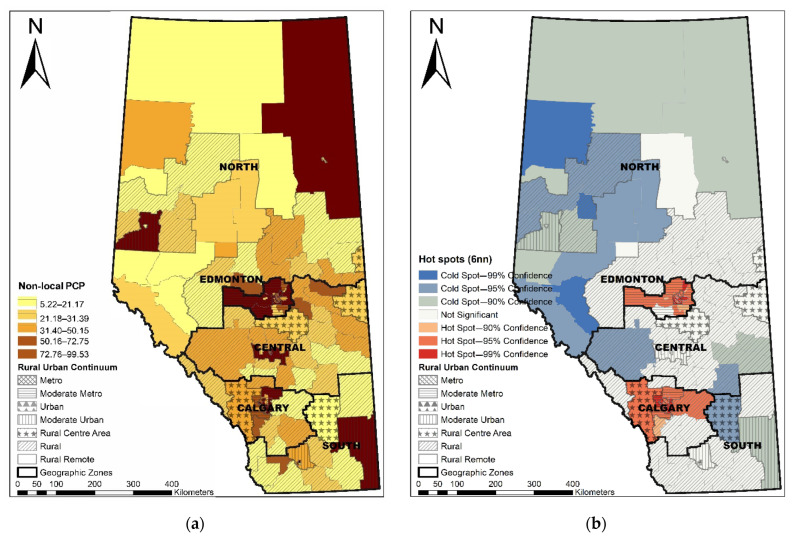
Distribution of non-local PCP utilization (**a**) and hot spots and cold spots of non-local PCP utilization (**b**) in Alberta.

With a spatial weights matrix defined by six nearest neighbors, the Moran’s I index is 0.38 (Z = 8.81, *p* < 0.001), suggesting a significant spatial pattern of clustering in non-local PCP utilization. Hot spot analysis identified hot spots with a high value of non-local PCP utilization in Edmonton and Calgary Zones and cold spots mostly in the northern remote areas (Figure 2).

### 3.2. Multivariate Linear Regression Models

As shown in Table 2, ACSC is highly correlated with the percentage of Aboriginal population (correlation coefficient: 0.70) and the rate of people with three and more comorbidities (correlation coefficient: 0.79). To avoid multicollinearity, we built the initial model with two different sets of independent variables: one set without the variables of aboriginal population and multimorbidity and the other set without ACSC. With a backward stepwise regression approach, both initial models resulted in the same final model. The final Alberta regression model (adjusted R^2^ = 0.71) included median travel time of patients with OA residing in a LGA, percentage of low-income families, and rural–urban residence, all of which were positively associated with non-local PCP utilization (Table 3). The results showed that LGAs with a longer median travel time to PCP and a higher percentage for the low-income family variable tended to utilize more non-local primary care. The non-local PCP utilization in broad urban areas was 29% higher than the broad rural areas while holding other significant variables constant in the model.

Two subset linear regression models were applied to the broad rural and urban LGAs (71 and 61), respectively, excluding the geographic variable rural–urban residence in the analysis. The two subset models identified the same significant variables (travel time and percentage of low-income families) as the Alberta model (Table 3) although the Alberta and rural models explained 72% of the total variance in non-local PCP utilization (adjusted R^2^ = 0.72) compared to an adjusted R^2^ of 0.45 in the urban model.

The model diagnostics suggested that the Alberta model violated the model assumption about the independent and identical distribution of residuals. Though the residuals from the Alberta model did not have significant spatial autocorrelation (Moran’s I = −0.03, *p* = 0.69), the residuals presented heteroskedasticity (Breusch–Pagan test 24.63, *p* = 0) and did not follow a normal distribution (Shapiro–Wilk test 0.97, *p* = 0.006). Further analysis at the provincial level is required to address this issue. Unlike the Alberta model, the residuals of both subset models were distributed normally and homogeneously.

### 3.3. Geographically Weighted Regression

As a GWR model already limits the spatial interaction among LGAs based on their spatial locations, the geographic variable rural–urban residence was excluded for spatial regression modeling. The presence of heteroskedasticity in the residuals of the Alberta linear regression model necessitates a GWR model, which addresses spatial non-stationarity in the pattern of non-local PCP utilization. The optimum neighbor size of 62 LGAs were used for each local regression. The low VIFs for each independent variable suggest there is negligible collinearity as no value exceeds two for any of the factors. As shown in Table 3, the GWR model identified three additional significant variables (ACSC, discontinuity of care, and percentage of people with a university degree) in addition to the two significant variables (median travel time of OA patients within an LGA and percentage of low-income families) identified with the Alberta regression model. Comparing the global linear regression model with the same formula as the GWR, the GWR model provided a better performance due to higher R^2^ (0.63 vs. 0.60), lower AIC (1110.03 vs. 1115.30), and a lower standard error in the residuals (16.41 vs. 16.58). Though the improvement of GWR over traditional linear regression was not substantial, the most important point was that the GWR model met the model assumption, while the global linear regression violated model assumptions due to the presence of heteroskedasticity and non-normal distributions.

**Figure 3:**
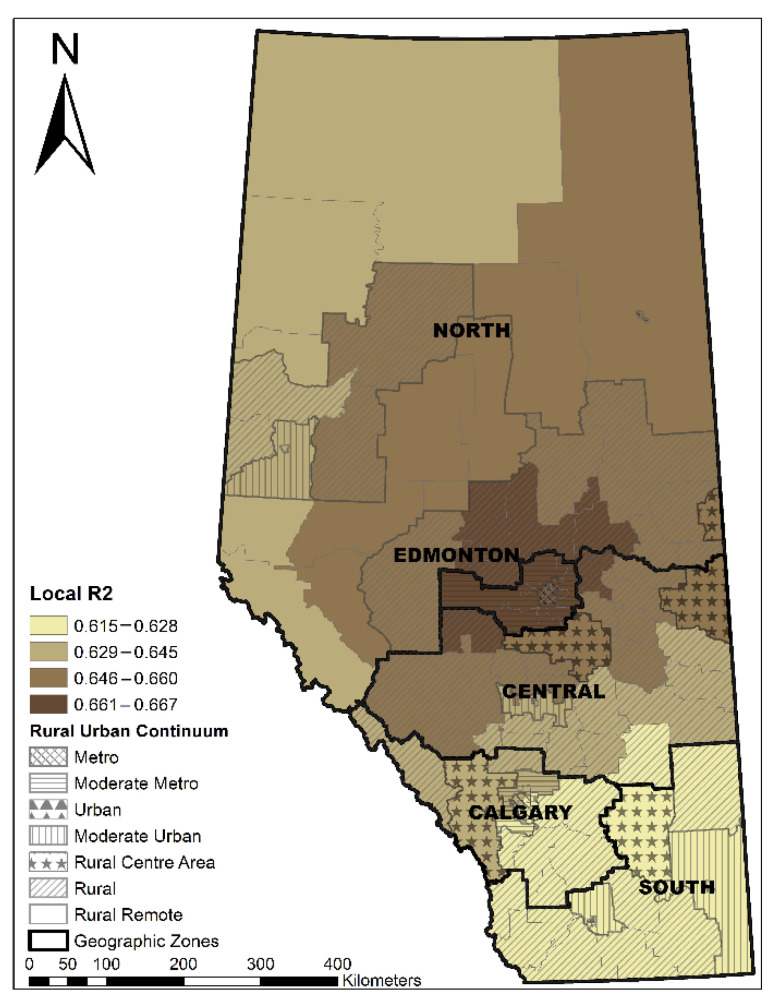
Distribution of local R^2^ of the GWR model.

**Figure 4:**
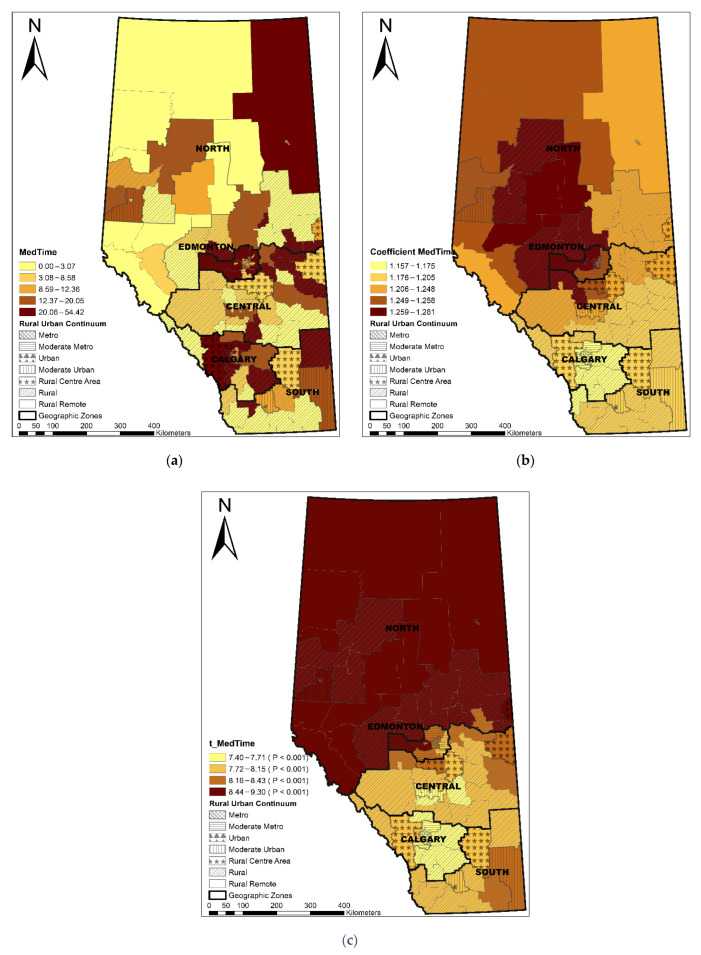
Distribution of median travel time (**a**), coefficient of median travel time (**b**), and significance of coefficient (**c**).

**Figure 5:**
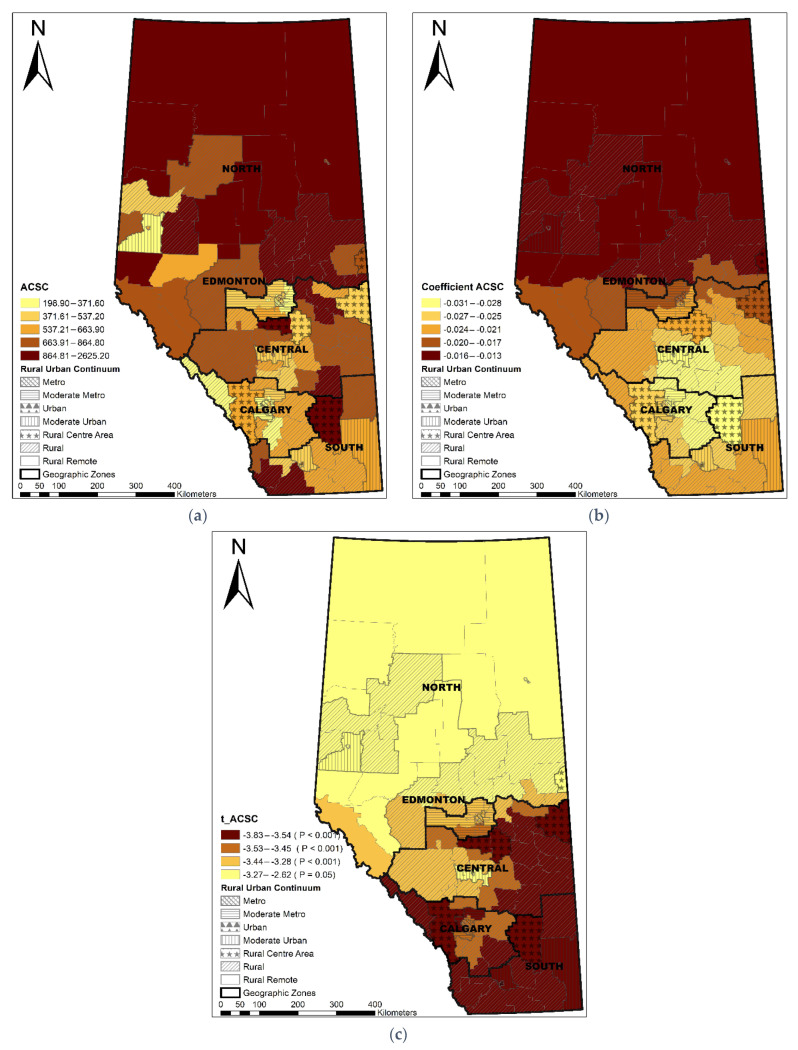
Distribution of ACSC (**a**), coefficient of ACSC (**b**), and significance of coefficient (**c**).

**Figure 6:**
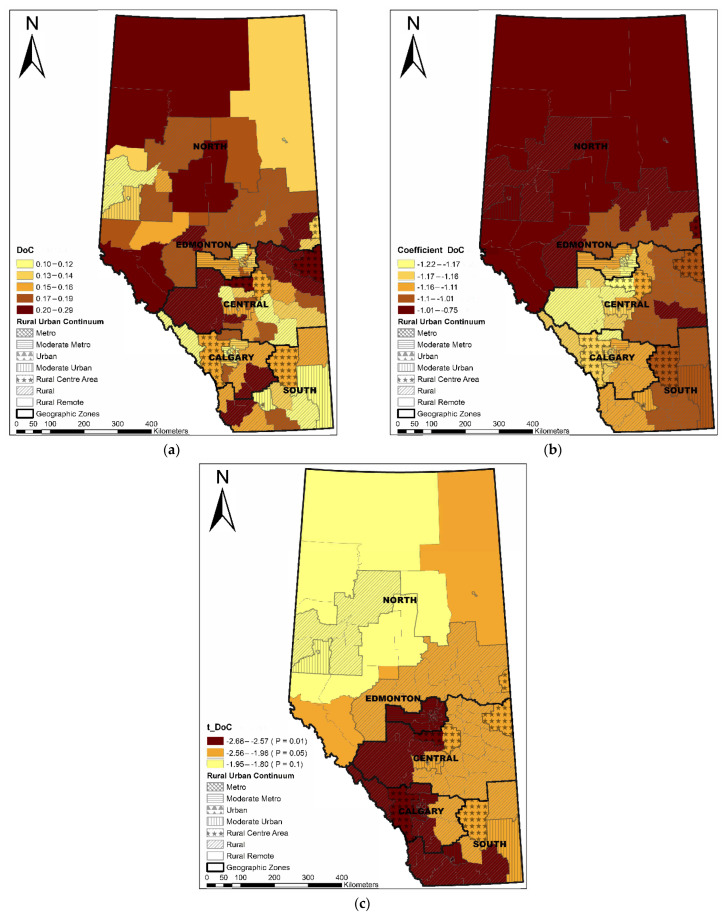
Distribution of discontinuity of care (**a**), coefficient of continuity of care (**b**), and significance of coefficient (**c**).

**Figure 7:**
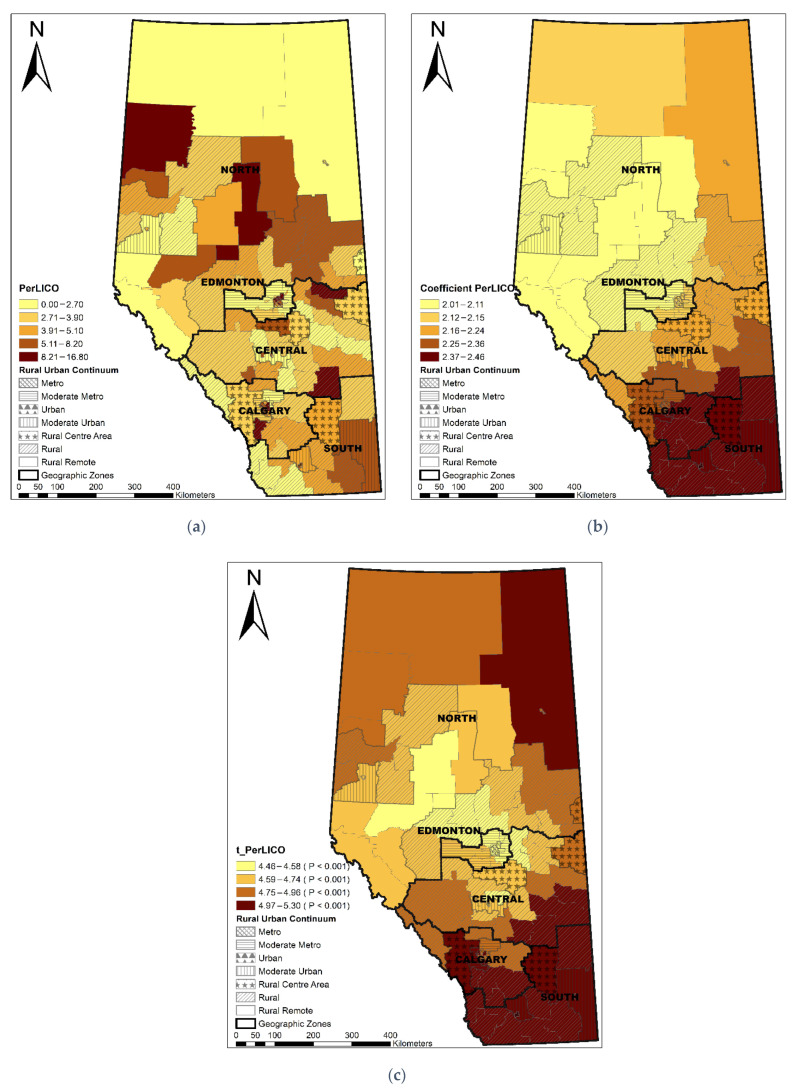
Distribution of percentage of low-income families (**a**), coefficient of percentage of low-income families (**b**), and significance of coefficient (**c**).

**Figure 8:**
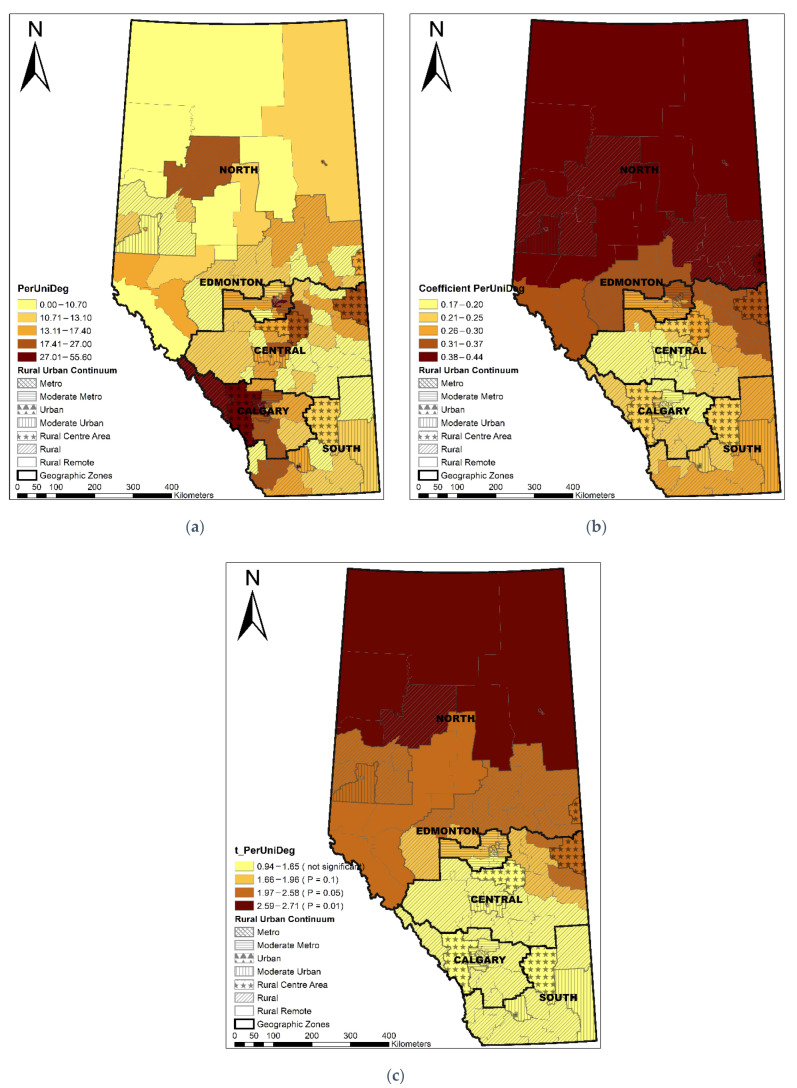
Distribution of percentage of people with university degrees (**a**), coefficient of percentage of university degrees (**b**), and significance of coefficient (**c**).

The Local R^2^ varied across the province, showing that the GWR model performed best in the Edmonton area, following a decreasing pattern towards the north and south areas (Figure 3). LGAs with a longer median travel time were significantly associated with a higher percentage of non-local PCP utilization (coefficient range: 1.16–1.28; *t* value range: 7.40–9.30) (Figure 4). ACSC had a significant negative association with non-local PCP utilization (coefficient range: −0.03–−0.01, *t* value range: −3.83–−2.62) (Figure 5). The discontinuity of care presented a negative association with non-local PCP visits. However, the significance of this association was not consistent across the province. As shown in Figure 6, discontinuity of care was most significant in the southwest of the province and not significant in the northern rural and remote areas. The percentage of low-income families was positively associated with the percentage of non-local visits (coefficient range: 2.01–2.46; *t* value range: 4.46–5.30) although the association was strongest in the south compared to the north zone (Figure 7). LGAs with a higher proportion of people with university degrees were associated with a higher non-local PCP utilization. The positive association was only significant in the north zone (Figure 8).

## 4. Discussion

In this study, we explored the association of area-level factors to the utilization of non-local PCP utilization at provincial and broad rural and broad urban areas, respectively, and examined if the association varied between rural and urban areas. Using multivariate linear regression, we identified that the median travel time within an LGA and the percentage of low-income families were significantly associated with the non-local PCP utilization in Alberta (adjusted R^2^: 0.71, including rural–urban residence), broad rural (adjusted R^2^: 0.72), and broad urban areas (adjusted R^2^: 0.45). The GWR model identified three additional significant variables (ACSC, discontinuity of care, and the percentage of people with a university degree). The rural–urban residence variable played a statistically significant role on the pattern of non-local PCP utilization.

We observed that the broad urban patients utilized 61% non-local PCP care compared to 26% in the broad rural areas. Hot spots of high non-local PCP utilization were identified in metro Edmonton and metro Calgary areas, while cold spots of low non-local PCP utilization were identified in rural remote areas. The behavior of patients with OA seeking non-local PCP care may be explained differently in rural and urban areas given the distribution of patients and providers and the difference in geographic characteristics. Our previous study identified that 50% of the prevalent OA cases resided in metro Calgary and Edmonton compared to 2.3% in rural remotes areas [57]. Of the 2312 PCP clinics, 54% are in metro Calgary and Edmonton compared to 2% in rural remote areas [33]. Furthermore, the size of 132 LGAs varies greatly across the province. The average size of 31 metro LGAs in Calgary and Edmonton is 50 km^2^ compared to the average size of 26,742 km^2^ in the 12 rural remote LGAs in the north zone [3]. As urban areas have a larger number of PCP options available within a highly populated, smaller geographic area, urban patients usually have reasonable access to PCPs across many LGAs. It is expected to see urban patients travelling anywhere within the urban areas, which may have minimal implications for the planning of PCP services in urban settings. This is not the case in rural areas. Conversely, rural patients are sparsely distributed in the vast rural local geographic areas, often having to travel far longer than their urban counterparts to visit a PCP even within the same LGA. Traveling outside local LGA indicates gaps in access to primary health in rural areas and is an important factor for planning PCP services in rural settings.

The median travel time of patients with OA to visit a PCP care was identified as a significant positive factor affecting the utilization of non-local PCP care at LGA level; that is, LGAs where patients travelled further to visit PCP had a higher percentage of non-local PCP visits. Patients in metro areas took 13 min (median travel time) to visit a PCP and had a higher percentage of non-local PCP visits across densely populated small urban LGAs, which is four times longer than their rural remote counterparts (median: 3 min) [33]. However, it is important to consider the varying travel behaviors for patients living in the vast, isolated rural communities. Though approximately 50% of the rural–remote OA patients visited a PCP within their local postal code (travel time = 0), about 30% of rural–remote patients visited non-local PCP care and took more than 60 min to access PCPs (95% percentile: 362 min) [33]. Further study focusing on local areas with high non-local PCP utilization and longer travel time to PCPs is of interest, as it may help policy makers and health care planners identify gaps in the supply of local primary health services. Patients travelled longer for non-local PCP care, especially in rural and remote areas, may be explained by the fact that there was no provision of primary care in their local community or because patients preferred services located outside of their local areas. Although the reasons remain uncertain, providing the right mix of local care with a variety of health care delivery options is key to meet the health care needs of local community populations that are being served. The right mix of care might include collaborative care pathways involving primary care physicians and allied health providers, namely physiotherapists, who can offer care to patients with OA regardless of their disease severity and consult readily with an orthopedic surgeon (virtually or otherwise) [58].

Numerous studies reported the association between neighborhood socioeconomic status and OA prevalence [24,25,59,60,61]. Compared to those with higher socioeconomic status, lower socioeconomic status is associated with higher use of hospitals, PCP services, and medications [62,63,64]. In this study, we identified that LGAs with a higher proportion of low-income families were associated with higher non-local PCP utilization. As shown in Figure 7, generally, metro and rural–remote LGAs had a higher percentage of low-income families than the rest of Alberta. Due to availability of clustered PCPs in metro areas and the small size of metro LGAs, metro patients may visit PCPs across LGAs without extra effort. While in rural–remote areas, patients may need to travel outside of local areas for PCP care as provision and capacity of primary care within local areas may not meet patients’ needs. Other factors such as patient adherence to treatment, patient attachment to PCP, level of provider continuity, and clinic capacity may affect the utilization of non-local care, which is beyond the scope of this analysis.

The results of this study could be applied to highlight factors that influence care outside of local areas, especially in rural and remote areas, informing health policy makers to address gaps in access to PCP care that has been measured by AH [11]. Both the GWR and multivariate linear regression models identified two factors (median travel time to PCP and the percentage of low-income families within an LGA) that were significantly associated with non-local PCP care at the provincial level. However, as shown by the GWR model, the discontinuity of care index and the percentage of population with a university degree were only significant in some local areas. Targeting provincial level factors such as travel time for example, common strategies on improving health care may be effective in both rural and urban settings. As OA patients usually have complex health needs due to aging populations, high prevalence of comorbidities, and reduced mobility, it is important to provide targeted appropriate options for health care delivery considering limited health care resources, gaps in access to PCP care, supply and distribution of PCPs, and other services to support the specific needs of OA patients. For example, the COVID-19 pandemic has demonstrated the important role of virtual care in supporting patients’ access to PCP care in a way that complements the in-person delivery of PCP services. At the local level, primary care networks are created through an agreement between primary care physicians and AHS that follow a team-based health care model where patients are paneled to a provider and clinic with a common-shared electronic medical record computer system to provide integrated care for all primary health care needs [65,66]. Furthermore, the association between health care utilization and socioeconomic status necessitates the system-level coordinated support by strengthening partnerships between health and social services. AHS developed the initiative of enhancing care in the community to achieve better health outcomes and improved patients experience through collaborations with communities, government departments, municipalities, and primary care providers. A renewed emphasis on virtual health services/telehealth and patients’ medical homes all features prominently in the recently published report on implementing high-quality primary care. Moreover, the report also addresses concern over the social determinants of health, unequal access, and health equity [67].

There are several strengths to our study. First, using provincial administrative databases as data sources, we identified a large OA cohort including 359,638 adult patients at the provincial level. Second, we obtained patient and provider’s clinic address at the six-digit postal code level from the administrative dataset. This is the finest spatial scale available for calculating travel times from a patient’s residential postal code to a provider’s clinic. Third, the analysis on the association between area-level factors and health care utilization is informative and could be applied to identify the factors that influence care outside of local areas. The findings will assist health planners and health service delivery to enhance care in the local community by developing policy and initiatives either at the local or provincial level.

We acknowledge the following limitations of this study. First, this finding of this study is more appliable in rural settings compared to the urban settings as seeking care outside local areas is of importance for rural planning, which may have minimal implications for urban planning. Second, the travel time was calculated between the population weighted centroids of two postal codes instead of the geocoded residential address. However, due to privacy and confidentiality, the six-digit postal code at the patient level is the most detailed spatial scale available for spatial analysis. Third, we used LGAs as the area unit to aggregate the utilization of non-local PCP care, which is subject to the modifiable areal unit problem (MAUP) and ecological fallacy [68]. The observed pattern of non-local PCP utilization and the reported associations in this study can only be interpreted at the LGA level. Due to the ecological fallacy, we are not able to deduce inferences at individual level, as our results only support inferences at the aggregated LGA level. However, the advantage of using LGA, an administrative geographic unit, as the area unit for analysis is that it aligns with the planning purposes of AHS, which uses LGA to monitor, plan, and evaluate the delivery of health services in Alberta. The sizes of LGAs vary greatly across rural and urban areas, ranging from 7 km^2^ in the metro areas to 99,994 km^2^ in the rural remote areas [3]. This may bring in bias when measuring travel time and cross-boundary travel patterns. For example, patients might be geographically located closer to a provider in another LGA if they live near the edge of their home LGA. Further study using a different spatial unit, for example, a travel-time-based buffer around a patient’s home, may be conducted to further understand the pattern of PCP utilization. Fourth, this study focused on the observed pattern of non-local PCP utilization and therefore did not account for patient attachment to a PCP, physician capacity, and whether a patient visited a PCP from work or home. These are challenges associated with the use of administrative data due to privacy and confidentiality. However, the use of administrative data provides a large dataset about the population of interest, which is already collected, enabling the analysis of utilization patterns at the population level. Last, the findings of this study provide insights to current health care delivery because of the shortage of physician services in rural and remote areas within Alberta in recent years. This study could be applied to identify the factors that influence care outside local areas, providing evidence for health care planners to address gaps in access to PCP services in rural and remote areas.

## 5. Conclusions

In conclusion, we examined the geospatial pattern of non-local PCP utilization and identified that travel time and income factors were significantly associated with PCP utilization in both rural and urban areas, while discontinuity of care and education attainment showed a geographically varying association with PCP utilization at local level. The results of this study could be applied to highlight factors that influence care outside of local areas, especially in rural and remote areas, informing health policy makers to identify gaps in access to PCP care, target policies at associated factors, and reduce rural–urban disparities in primary care provision.

## Figures and Tables

**Table 1:** List of response and predictor variables at LGA level.

Variables		Definition	Sources
Response variable	Non-local PCP utilization	Percentage of PCP visits outside patients’ local LGA (outside visits/total visits of LGA*100)	Alberta Health (AH) Physician Claims
Predisposing factors	MedAge2013	Median age of OA patients visiting PCP in 2012/2013	AH Population Registry and AH Physician Claims
	Per65Lone	Percentage of 65 years of age and older who live alone	Alberta Health Primary Health Care—Community Profiles 2013. Data sources are Statistics Canada Federal Census and Alberta Health Population Registry.
	PerFemale	Percentage of female patients among total OA patients visiting PCP (number of females/total patients visiting PCP*100)	AH Population Registry and AH Physician Claims
	PerLoneFemale	Percentage of female lone-parent families	Alberta Health Primary Health Care—Community Profiles 2013. Data sources are Statistics Canada Federal Census and Alberta Health Population Registry.
	PerImmig	Percentage of immigrants who arrived in the last five years	Alberta Health Primary Health Care—Community Profiles 2013. Data sources are Statistics Canada Federal Census and Alberta Health Population Registry.
	PerAborig	Percentage of First Nations with treaty status and Inuit	Alberta Health Primary Health Care—Community Profiles 2013. Data sources are Statistics Canada Federal Census and Alberta Health Population Registry.
Enabling factors	MedTime	Median travel time of PCP visits	AH Population Registry and AH Physician Claims
	ACSC	Ambulatory care sensitive conditions, age-standardized separation rate per 100,000 population	Alberta Health Primary Health Care—Community Profiles 2013. Data source is ambulatory care data.
	DoC	Discontinuity of care index. Percentage of patients that have chronic conditions but do not have a PCP visits within a three-year timeframe over the total general population	Alberta Health Primary Health Care—Community Profiles 2013. Data sources are Physician Claims and Alberta Health Population Registry.
	PerUniDeg	Percentage of population with university certificate, diploma, or degree	Alberta Health Primary Health Care—Community Profiles 2013. Data sources are Statistics Canada Federal Census and Alberta Health Population Registry.
	PerLICO	Percentage of families with after-tax low-income	Alberta Health Primary Health Care—Community Profiles 2013. Data sources are Statistics Canada Federal Census and Alberta Health Population Registry.
	AvgFIncome	Average census family income	Alberta Health Primary Health Care—Community Profiles 2013. Data sources are Statistics Canada Federal Census and Alberta Health Population Registry.
	Rurban_2cat	Broad rural vs. broad urban	Alberta Standard Geographic Areas
Needs	CruRateOA	Crude rate of people with OA among general population (Registry) (per 100 population)	AH Population Registry and AH Physician Claims
	CmbOver3Rate	Age-standardized rate of people with three and more comorbidities among general population (per 100 population)	Alberta Health Primary Health Care—Community Profiles 2013. Data sources are Population Registry and Physician Claims.

**Table 2:** Correlation coefficients between variables.

	Non-Local PCP	MedTime	MedAge2013	PerFemale	PerAborig	PerLoneFemale	Per65Lone	PerLICO	AvgFIncome	PerImmig	PerUniDeg	ACSC	CmbOver3Rate	DoC	CruRateOA	Rurban_2cat
**Non-local PCP**	1	0.55	−0.10	0.12	−0.15	0.13	−0.14	0.26	0.30	0.38	0.46	−0.44	−0.23	−0.36	−0.29	0.70
**MedTime**	0.55	1	−0.15	−0.19	0.06	−0.19	−0.12	−0.16	0.17	−0.04	0.10	−0.15	−0.08	−0.10	0.06	0.16
**MedAge2013**	−0.10	−0.15	1	0.40	−0.45	−0.16	0.32	0.11	−0.14	−0.02	0.15	−0.20	−0.25	0.06	0.30	−0.09
**PerFemale**	0.12	−0.19	0.40	1	−0.16	0.24	−0.05	0.37	0.05	0.39	0.28	−0.11	−0.05	−0.19	−0.15	0.24
**PerAborig**	−0.15	0.06	−0.45	−0.16	1	0.37	−0.14	−0.03	−0.24	−0.22	−0.22	0.70	0.60	0.20	0.19	−0.30
**PerLoneFemale**	0.13	−0.19	−0.16	0.24	0.37	1	0.04	0.60	−0.29	0.24	0.05	0.26	0.45	−0.01	−0.08	0.19
**Per65Lone**	−0.14	−0.12	0.32	−0.05	−0.14	0.04	1	0.05	−0.19	0.02	0.08	−0.02	−0.08	0.12	0.16	−0.08
**PerLICO**	0.26	−0.16	0.11	0.37	−0.03	0.60	0.05	1	−0.27	0.52	0.17	−0.01	0.19	−0.05	−0.10	0.27
**AvgFIncome**	0.30	0.17	−0.14	0.05	−0.24	−0.29	−0.19	−0.27	1	0.19	0.63	−0.46	−0.48	−0.41	−0.54	0.44
**PerImmig**	0.38	−0.04	−0.02	0.39	−0.22	0.24	0.02	0.52	0.19	1	0.59	−0.39	−0.26	−0.33	−0.45	0.49
**PerUniDeg**	0.46	0.10	0.15	0.28	−0.22	0.05	0.08	0.17	0.63	0.59	1	−0.56	−0.56	−0.41	−0.44	0.55
**ACSC**	−0.44	−0.15	−0.20	−0.11	0.70	0.26	−0.02	−0.01	−0.46	−0.39	−0.56	1	0.79	0.35	0.39	−0.61
**CmbOver3Rate**	−0.23	−0.08	−0.25	−0.05	0.60	0.45	−0.08	0.19	−0.48	−0.26	−0.56	0.79	1	0.38	0.33	−0.35
**DoC**	−0.36	−0.10	0.06	−0.19	0.20	−0.01	0.12	−0.05	−0.41	−0.33	−0.41	0.35	0.38	1	0.33	−0.53
**CruRateOA**	−0.29	0.06	0.30	−0.15	0.19	−0.08	0.16	−0.10	−0.54	−0.45	−0.44	0.39	0.33	0.33	1	−0.43
**Rurban_2cat**	0.70	0.16	−0.09	0.24	−0.30	0.19	−0.08	0.27	0.44	0.49	0.55	−0.61	−0.35	−0.53	−0.43	1

**Table 3:** Multivariate linear regression and GWR model of non-local PCP utilization at LGA level.

Variables		Linear Regression (Alberta, 132 LGAs)		Linear Regression (Broad Rural, 71 LGAs)		Linear Regression (Broad Urban, 61 LGAs)		GWR Model		Global Linear Regression (No Rural–Urban)	
		Beta	*t* Value	Beta	*t* Value	Beta	*t* Value	Beta	*t* Value	Beta	*t* Value
Predisposing factors	MedTime	1.15	10.21	0.90	13.17	2.20	7.02	1.23	8.15	1.24	9.30
	ACSC							−0.02	−3.45	−0.02	−3.02
	DoC							−1.14	−2.59	−0.93	−2.35
	PerLICO	1.40	3.79	1.01	3.07	2.40	3.95	2.19	4.74	2.28	5.28
	PerUniDeg							0.27	1.62	0.39	2.48
	Rurban_2cat (Urban)	29.46	11.46	N/A	N/A	N/A	N/A	N/A		N/A	
Model diagnostic	**R^2^**	0.72		0.72		0.47		0.63		0.60	
	**Adj. R^2^**	0.71		0.72		0.45				0.59	
	**AIC**	1066.27		487.09		522.72		1110.03		1115.30	
	**Res.SE**	13.85		7.58		16.87		16.41		16.58	
	**Shapiro–Wilk test**	0.97, *p* = 0.006		0.98, *p* = 0.68		0.99, *p* = 0.99		0.98, *p*= 0.15		0.98, *p* = 0.052	
	**Breusch–Pagan test**	24.63, df = 3, *p* = 0		3.02, df = 2, *p* = 0.22		3.33, df = 2, *p* = 0.19		N/A		15.95, df = 5, *p* = 0.007	
	**Moran’s I test**	−0.03, *p* = 0.69		N/A		N/A		0.02, *p* = 0.56		−0.007, *p* = 0.45	

**Figure A1:**
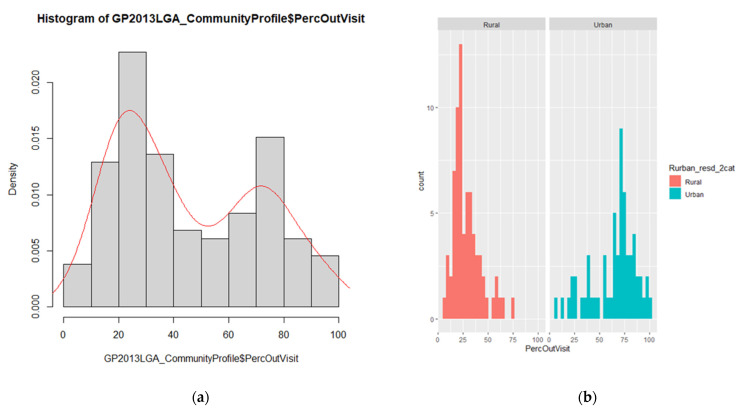

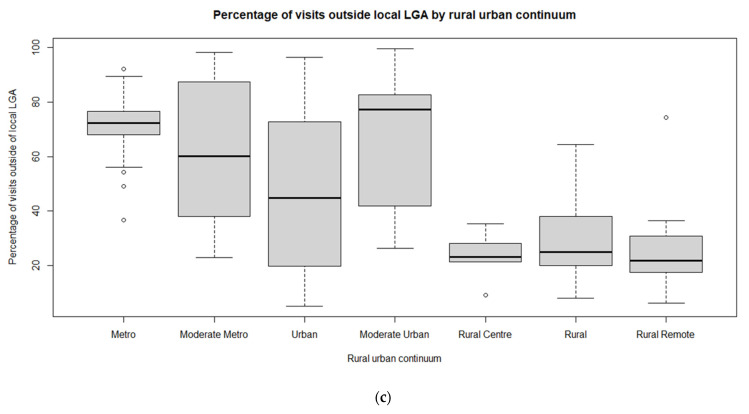
Histograms of non-local PCP utilization at provincial level (**a**), broad rural and urban area level (**b**), and boxplots of non-local PCP utilization at rural–urban continuum level (**c**).

## Data Availability

Restrictions apply to the availability of these data. Data was obtained from Alberta Health Services and are available from the corresponding author with the permission of Alberta Health Services.
